# Mechanism of DNA Lesion Homing and Recognition by the Uvr Nucleotide Excision Repair System

**DOI:** 10.34133/2019/5641746

**Published:** 2019-08-28

**Authors:** Seung-Joo Lee, Rou-Jia Sung, Gregory L. Verdine

**Affiliations:** ^1^Department of Stem Cell and Regenerative Biology, Harvard University, Cambridge, MA 02138, USA; ^2^Department of Molecular and Cellular Biology, Harvard University, Cambridge, MA 02138, USA; ^3^Department of Chemistry and Chemical Biology, Harvard University, Cambridge, MA 02138, USA

## Abstract

Nucleotide excision repair (NER) is an essential DNA repair system distinguished from other such systems by its extraordinary versatility. NER removes a wide variety of structurally dissimilar lesions having only their bulkiness in common. NER can also repair several less bulky nucleobase lesions, such as 8-oxoguanine. Thus, how a single DNA repair system distinguishes such a diverse array of structurally divergent lesions from undamaged DNA has been one of the great unsolved mysteries in the field of genome maintenance. Here we employ a synthetic crystallography approach to obtain crystal structures of the pivotal NER enzyme UvrB in complex with duplex DNA, trapped at the stage of lesion-recognition. These structures coupled with biochemical studies suggest that UvrB integrates the ATPase-dependent helicase/translocase and lesion-recognition activities. Our work also conclusively establishes the identity of the lesion-containing strand and provides a compelling insight to how UvrB recognizes a diverse array of DNA lesions.

## 1. Introduction

The genomes of all organisms are vulnerable to attack by a wide variety of DNA-damaging agents of both endogenous and environmental origins. The lesions resulting from these attacks alter the covalent structure of DNA, thereby causing mutations that in prokaryotes decrease viability and in differentiated eukaryotes give rise to cancer [1, 2]. The immense structural diversity of these lesions poses a challenge to the DNA repair systems that have evolved to protect genomes from genotoxic damage. The base excision DNA repair system (BER) solves this problem by elaborating an ensemble of highly lesion-specific repair enzymes, whereas the nucleotide excision DNA repair pathway (NER) employs a single damage-detection and damage-eradication system to process a broad array of structurally dissimilar lesions. NER was first discovered in prokaryotes for its ability to protect against the mutagenic and toxic effects of ultraviolet (UV) radiation [3, 4]. Resistance to UV was found to result from enzymatic removal of UV-induced photoproducts, namely, cyclobutane pyrimidine dimers (CPD) and 6-4 photoproducts, through the coordinated action of UvrA, UvrB, and UvrC [5]. The Uvr system was subsequently found to be responsible for removal of many different genotoxic lesions, which bore little if anything in common saves for their possession of a bulky structure [6]. Whereas the exquisite lesion-selectivity of BER enzymes could readily be understood in structural terms, the ability to distinguish lesions from undamaged DNA merely on the basis of bulkiness has been difficult to rationalize. Adding further to the mystery is the fact that the Uvr system also accepts as substrates a number of nonbulky lesions, including 8-oxoguanine and thymine glycol [7–9]. Studies of prokaryotic NER have proven paradigmatic for understanding the much more elaborate eukaryotic pathway. In humans, the components of NER were largely discovered and elucidated through their mutational inactivation having given rise to numerous serious autosomal recessive diseases [10].

**Figure 1:**
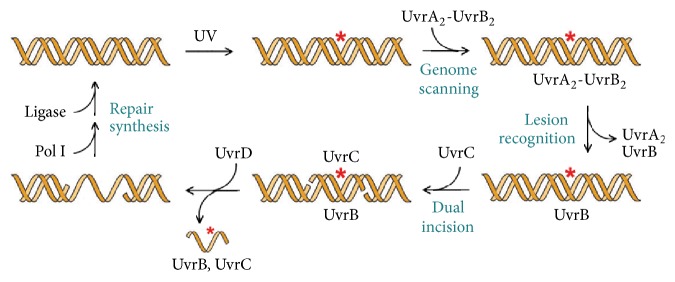
Schematic of Uvr nucleotide excision repair pathway.

Lesion recognition via the Uvr pathway is initiated by the UvrA_2_B_2_ complex, which scans the genome for lesions (Figure 1) [11, 12]. Within this complex, UvrA acts as the initial damage sensor but hands off the lesion-containing DNA to UvrB and dissociates, leaving UvrB bound to the lesion in a preincision complex, the precise nature of which is not known. Both UvrA and UvrB expend ATP to form the preincision complex, which is believed to require threading of a *β*-hairpin element on UvrB through the DNA duplex [13]. UvrB presents the lesion-containing strand to UvrC, which catalyzes hydrolysis of the 5th phosphodiester bond 3′ to the lesion and then the 8th phosphodiester bond 5′ to the lesion [6]. Crystal structures have begun to yield a molecular-level understanding of the Uvr pathway [12, 14–20]; however, the reductionist and fragmentary nature of these structures leave many fundamental questions unanswered, such as which DNA strand bound to UvrB contains the lesion, and of course how UvrB recognizes such an eclectic assortment of lesions.

Here we present the first crystal structure of a trapped UvrB-DNA preincision complex. This structure plus supporting biochemical studies elucidates how UvrB homes in on lesions and discriminates damaged sites from undamaged ones.

## 2. Results

### 2.1. Trapping a Preincision Complex

**Figure 2:**
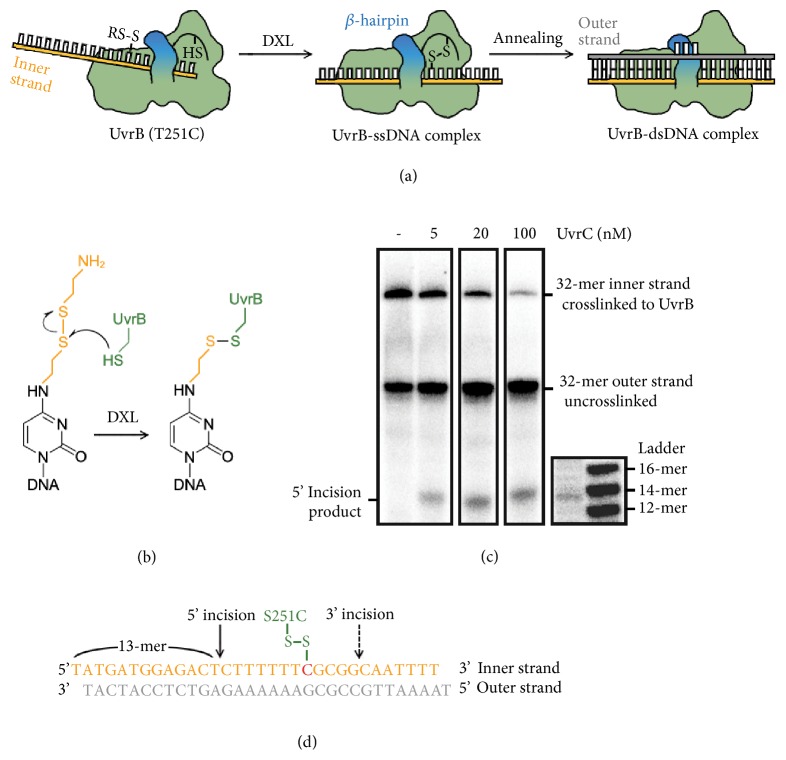
Entrapment strategy to form a preincision complex. (a) Schematic describing the disulfide crosslinking (DXL) and stepwise assembly strategy. (b) Details of the trapping chemistry. A cysteine residue engineered into UvrB attacks a disulfide-bearing tether attached to the N^4^-position of a cytosine. Curved arrows denote electron flow in the crosslinking reaction. (c) UvrC incision assays on the crosslinked UvrB-dsDNA complexes were analyzed on 15% denaturing polyacrylamide gel. 5′ ends of both inner and outer strands are radioactively labeled. Lanes 1-4 were cropped from the same experiment. Lanes 5 and 6 (with DNA ladder) were run on 23.5% denaturing polyacrylamide gels for better resolution. (d) DNA sequence used for this study and experimentally determined 5′ and inferred 3′ incision sites. The lesion-mimetic cytosine, located 8-nucleotide downstream on the inner strand, is shown in red.

Previous crystallographic and biochemical studies have suggested that the exceptional stability of UvrB preincision complexes results from insertion of a flexible *β*-hairpin element between the two DNA strands at an as-yet undetermined locus within a ~6 base-pair (bp) unpaired duplex segment “bubble” containing the lesion [6]. Under physiological conditions, the energetically demanding process of wresting open duplex DNA at the lesion and inserting the *β*-hairpin is fueled by ATP hydrolysis by both UvrA and UvrB [21]. The same energy-intensive conditions that load UvrB onto a lesion promote dissociation of the two (vide infra), and this has thwarted all previous attempts to crystallize a UvrB preincision complex. We reasoned that it might be possible to synthesize a UvrB preincision complex through a stepwise assembly process that would eliminate the need for ATP expenditure and kinetically trap the complex from dissociation, while restricting the roaming range of UvrB on DNA. In previous studies, we [22–25] and others [26, 27] have employed a synthetic crystallography approach centered upon the ability of intermolecular disulfide-crosslinking (DXL) to trap otherwise transient intermediates in protein-DNA recognition [28]. Our studies build upon the groundbreaking work of van Houten, Kisker, and Goosen, which revealed that UvrB extrudes from the DNA a nucleoside in the strand clamped by the *β*-hairpin loop (hereafter called ‘inner' strand) [17, 29]; the extruded nucleobase inserts into the highly conserved hydrophobic pocket. We therefore introduced a mutant Cys residue into the hydrophobic pocket and disulfide-crosslinked the resulting UvrB(T251C) protein to a single-stranded DNA substrate containing a thiol-tethered nucleobase, a cytosine. We purified this species by ion exchange and then annealed the complementary strand (the outer strand) to produce UvrB bound to duplex DNA (Figures 2(a) and 2(b)). The complimentary strand is designed to contain two pyrimidine-pyrimidine mismatches on the immediate 5′ side of the modified cytosine; these were introduced to destabilize the duplex in the region expected to form a bubble, thereby facilitating maintenance of the helix-penetrated state for the *β*-hairpin. The corresponding DNA duplex containing no mismatched base-pairs failed to yield diffraction-quality crystals.

To establish the physiologic relevance of the preincision complex trapped by this approach, we subjected UvrB(T251C) synthetically bound to 5′ end labeled 32-mer DNA duplex to UvrC incision assay. Although incision efficiency is low, presumably because DNA tethering prevents UvrB from properly positioning incision sites for endonuclease activity, the UvrB-bound DNA duplex was recognized and acted upon by the downstream endonuclease UvrC, as judged by UvrC having made an ATP-dependent 5′-incision in the inner strand tethered to UvrB (Figure 2(c)). This experiment establishes that stepwise complex assembly governed by DXL yields a viable, physiologically relevant preincision complex. Furthermore, as incision is known to occur exclusively on the lesion-containing strand, this result shows definitively that the inner strand in our complex is bound in such a way as to mimic a bona fide lesion; hence the inner strand is the lesion-containing strand. Incision is known to take place at the 8th phosphodiester bond 5′-to the lesion, thus allowing us to infer that the enforced extrahelical cytosine in our structure is being presented by UvrB to UvrC as though it were a bona fide lesion, and UvrC is recognizing it as such (Figure 2(d)). Further biochemical evidence in favor of these assertions is given below.

### 2.2. Overall Structure of UvrB Bound to a Duplex DNA

**Figure 3:**
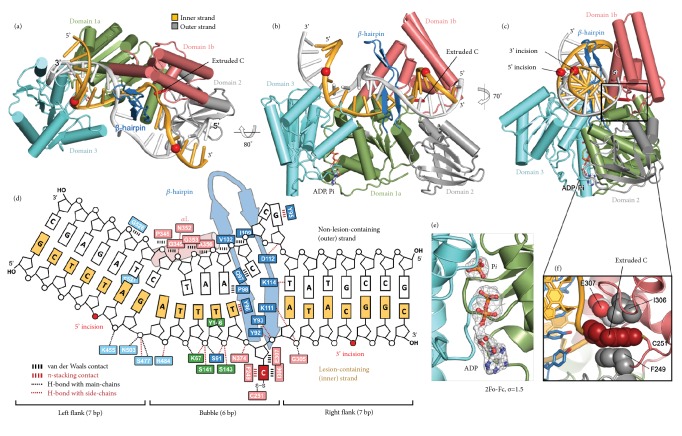
Overall structure of the preincision complex. (a, b, c) Structure of the UvrB-dsDNA-ADP·Pi complex in three views. UvrB domains 1a, 1b, 2, and 3 and the *β*-hairpin are colored in green, pink, grey, cyan, and blue, respectively. The crosslinked cytosine on the inner strand (gold) is highlighted in red. ADP and Pi are shown in sticks. Experimentally determined UvrC incision sites on the inner strand are shown as red spheres. (d) Schematic illustrating the interactions between UvrB and DNA. Colour-coding for UvrB residues is as in (a-c). (e) 2F_o_ – F_c_ electron density (grey mesh), contoured at 1.5s, of ADP and Pi. (f) Close-up view of the extrahelical cytosine embedded inside the narrow hydrophobic pocket.

UvrB is classified as a member of SF2 helicases on the basis of conserved helicase motifs (I-VI) [30] (Supplementary [Supplementary-material supplementary-material-1]). It contains the classical RecA-like domains (domains 1a and 3), in addition to three auxiliary domains (domains 1b, 2, and 4), plus the element aforementioned *β*-hairpin that projects from domain 1a. We determined crystal structures of UvrB disulfide-crosslinked to a 20-mer duplex substrate, in both the presence and absence of ATP, at 2.6 Å and 2.8 Å resolution, respectively (Figure 3 and [Supplementary-material supplementary-material-1]). The structures presented herein contain UvrB domains 1a through 3 (residues 1-593); domain 4, which lacks electron density in the previous structure due to structural polymorphism [16], was omitted for the structural studies but retained for biochemical experiments. For those crystals grown in the presence of ATP, we clearly observe a nucleotide bound to the ATP binding site located at the interdomain cleft within helicase domain. However, the electron density corresponds to ADP plus inorganic phosphate (Figure 3(e)); hence we conclude that the ATPase site retains activity in our crosslinked complex.

As expected on the basis of the synthetic crystallography approach used to trap these structures, the 13th nucleobase on the inner strand, a cytosine, is extruded from the duplex and inserted into the hydrophobic pocket between domains 1a and 1b (Figure 3(f)). As predicted, in our structures the *β*-hairpin loop was found to be fully inserted between the two DNA strands, clamping the inner strand between the *β*-hairpin and domain 1b (Figures 3(a)–3(c)). Though insertion of the *β*-hairpin loop requires disruption of only one or two base-pairs at the insertion site, our structures reveal disruption of base-pairing throughout the entire 6 bp duplex region comprising the crosslinked cytosine. This disruption of intact Watson-Crick base-pairs appears to be accomplished by UvrB-induced unwinding of the duplex substrate immediately upstream of the lesion. The size of the induced bubble observed in our complex is fully consistent with the bubble size as determined via biochemical experiments [31].

**Figure 4:**
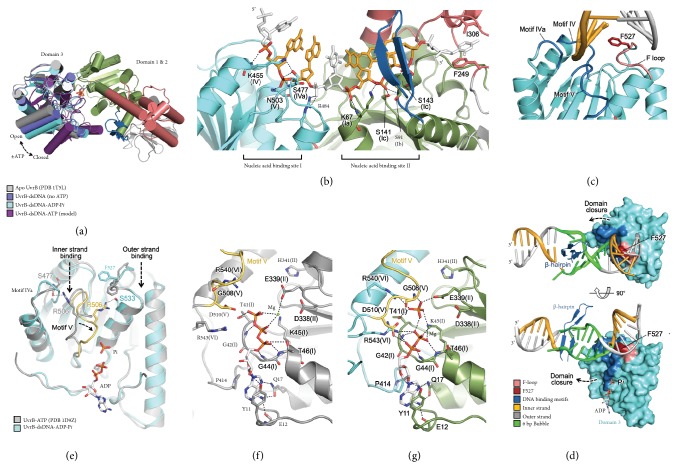
Proposed translocation mechanism of UvrB. (a) Proposed rigid body movement of RecA-like domains upon ATP-binding and ATP-hydrolysis. (b) Interactions between the inner strand and two nucleic acid binding sites in domains 1a and 3. For clarity, only 8-nucleotide stretch of the inner strand is shown. Nucleotides in contact with helicase motifs are shown in colors while others are shown in white. The Interactions are represented in dashed lines in all panels. (c) F527 (red) lodged deeply in the minor groove. Helicase motifs and F-loop are colored in blue and pink, respectively. (d) DNA bubble (green) flanked by F527 on one end and the *β*-hairpin on the other in two orthogonal views. Domains 1a, 1b, and 2 are omitted for clarity. (e) Superposition of domain 3 of UvrB in the absence (grey; PDB ID, 1D9Z) [16] and presence of dsDNA substrate (cyan). Motif V, which undergoes conformational transition upon DNA binding, is highlighted in yellow. (f) The ATPase site of UvrB bound to ATP in the absence of dsDNA (PDB ID, 1D9Z) [16]. (g) The activated ATPase site in the UvrB-dsDNA-ADP·Pi structure.

Previous DNA-bound structures of UvrB bore only fragmentary nucleic acids, leaving uncertain the overall disposition of the DNA duplex with respect to the protein. In our structures of a lesion-mimetic recognition complex, the duplex runs along the entire long axis of UvrB, contacting every domain except for domain 2 (Figures 3(a)–3(c)). The backbone phosphates of the inner DNA strand make extensive contacts to the domains 1a and 3 (Figures 3(d) and 4(b)). The outer strand also makes contacts to UvrB, but these comprise almost exclusively van der Waals interactions (Figure 3(d)). In agreement with previous EM studies [32], the UvrB-bound DNA contains a sharp bend (~60°) localized at the junction between the bubble and the fully base-paired segment flanking on its left side (“left flank,” Figure 3(d)). Bending appears to increase the accessibility of the 5′-incision site experimentally determined here, which is located on the left flank, in addition to drawing the 5′-incision site closer in space to the 3′-incision site (Figure 3(a)). Flanking the bubble and hairpin insertion site on the right side is the fully base-paired duplex segment “right flank” containing the inferred 3′-incision site (5th phosphodiester bond 3′ to the lesion); this too is almost completely exposed (Figure 3(c)). Thus, our structures reveal that UvrB grasps the duplex so as not only to recognize the lesion, but, as hypothetically proposed previously [33], also to present the scissile phosphates indeed on the same face of the DNA duplex and with optimal accessibility and geometry.

### 2.3. Interactions between DNA and the *β*-Hairpin Loop

The *β*-hairpin loop is a structural motif that is commonly employed by DNA helicases as a “pin” to separate two DNA strands [34–36]. In the current structures, we observed that the *β*-hairpin unwinds the duplex DNA in the 5′ to 3′ direction. The contacts between the *β*-hairpin loop and the inner strand are almost identical to the previously determined structure of UvrB bound to DNA containing a simple stem-loop structure [17]. The DNA in that structure contains a single-stranded extension projecting from the stem, and the first nucleoside of that extension adopts an extrahelical conformation and is inserted into the same slot on UvrB as is the extrahelical lesion-mimetic cytosine in our structures. A triad of three strictly conserved and functionally critical tyrosine residues located at the inner face of the *β*-hairpin (Y92, Y93, and Y96) occupy the space vacated by the extrahelical nucleoside; Y92 and Y93 form hydrogen bonds to phosphates neighboring the extruded nucleotide, and Y96 forms *π*-stacking interaction with the base directly 5′ to the extruded nucleotide on the inner strand (Figure 3(d) and Supplementary [Supplementary-material supplementary-material-1]). The extruded nucleobase undergoes ~90° rotation around its glycosidic bond to be fully embedded within the hydrophobic pocket constricted by type-conserved residues I306 and F249 (Figure 3(f)). With respect to the outer strand, helix penetration by the *β*-hairpin results in three extrahelical bases. The first two of them are stacked over Y95, which is also important for formation of a stable preincision complex [37].

### 2.4. Implications for UvrB Translocation Mechanism

Previous crystallographic studies on SF2 helicases demonstrated that ATP binding and hydrolysis are associated with rigid-body movements within the RecA-like domains, alternating between “open” and “closed” states and that this movement repositions two nucleic-acid binding surfaces, thereby driving translocation of the enzyme along the nucleic acid [38]. On the basis of its conserved helicase motifs, the RecA-like domains of UvrB (domains 1a and 3) are likely to undergo similar conformational changes in response to ATP-binding and ATP-hydrolysis. If so, the observed contacts between the helicase motifs engaged in DNA binding and the inner strand phosphates suggest that our structures obtained in both the presence and absence of ATP (hydrolyzed to ADP and Pi* in crystallo*) represent “open” states (Figure 4(a)). The hallmark of the open state is that, in it, the successive string of phosphates contacted by the two RecA-like domains is separated by one phosphate (Figure 4(b) and Supplementary Figures [Supplementary-material supplementary-material-1] and [Supplementary-material supplementary-material-1]) [34, 39], while in the closed state the respective string of phosphates forms a continuous array of contacts [39–41] (Supplementary [Supplementary-material supplementary-material-1]). Even though our structures represent the open state, we note that the domains 1a and 3 from these structures could be individually superimposed on their respective counterparts in closed structures of SF2 helicases with no major steric clash, suggesting that UvrB can also adopt the closed state on DNA (Figure 4(a)).

Unlike other helicases studied to date, UvrB catalyzes the translocation of a “bubble” along a stretch of otherwise fully paired duplex DNA, becoming positionally fixed only upon encounter with a lesion. This unique interaction mode of UvrB is apparently enabled by extensive protein contacts made to both strands of duplex DNA (Figure 3(d)). Of particular note is the loop preceding motif VI (F-loop, residues G526-S530) that approaches but does not snugly contact the outer strand at the left flank (Figure 4(c)). A key feature of the F-loop is the functionally critical residue F527 [33], which in our structure is lodged deeply in the minor groove, but not so deeply as to intercalate between DNA bases. If the UvrB helicase domain underwent rigid-body movements similar to those seen for other helicases as proposed previously [33], ATP-dependent domain closure would force the F-loop toward DNA (Figure 4(d)), perhaps causing F527 to intercalate into the duplex stack, which may function as a “ratchet” to facilitate unidirectional translocation upon ATP-hydrolysis. Indeed, in a structure we determined of UvrB (S91C) bound to a fully base-paired DNA duplex, F527 is intercalated into a sharply bent DNA duplex (Supplementary Figures [Supplementary-material supplementary-material-1] and [Supplementary-material supplementary-material-1]); furthermore, the crystal structure of the analogous complex containing a F527A mutation revealed substantially reduced DNA bending (Supplementary [Supplementary-material supplementary-material-1]) and increased DNA disorder presumably due to the loss of “ratchet” that seems to lock the duplex DNA in position (Supplementary Figures [Supplementary-material supplementary-material-1] and [Supplementary-material supplementary-material-1]). That F527 intercalation is important functionally suggested by the reduced incision efficiency observed for the F527A mutant UvrB [33].

### 2.5. DNA Binding Activates UvrB ATPase Activity

The ATPase activity of UvrB is known to be stimulated by DNA binding [42], and the present structures suggest a mechanism for such activation. Crystal structures of several related helicases have revealed that helicase motif V binds to both nucleic acid and ATP, thereby coordinating ATPase-driven translocation along the nucleic acid strand [39, 40]. However, in all UvrB structures lacking duplex DNA, R506 in motif V forms hydrogen bonds with S477 in motif IVa, resulting in disengagement of motif V from the ATP binding site (Figures 4(e) and 4(f)) [16, 17, 43]. In our structures, however, these interactions are disrupted by the inner DNA strand binding into the shallow depression formed between motifs IVa and V. This severs the connection between motifs IVa and V, releasing motif V to contact directly all three phosphate moieties of ATP (or of ADP and Pi) through the main chain of G508 and the side chain of D510, while simultaneously stabilizing the catalytically essential residues R540 and R543 of motif VI to form hydrogen bonds with the *β*- and *γ*-phosphates (Figures 4(e) and 4(g)). These newly observed contacts facilitate the proper positioning of the *γ*-phosphate with respect to E339 of the DExH motif (motif II), the side chain carboxylate of which is believed to activate a water molecule for nucleophilic attack on the *γ*-phosphate. Consistent with our observations, mutagenesis studies showed that all motifs V and VI residues mentioned above are critical for ATPase activity or the formation of a preincision complex or both [44, 45].

### 2.6. Identification of the Lesion-Containing Strand

**Figure 5:**
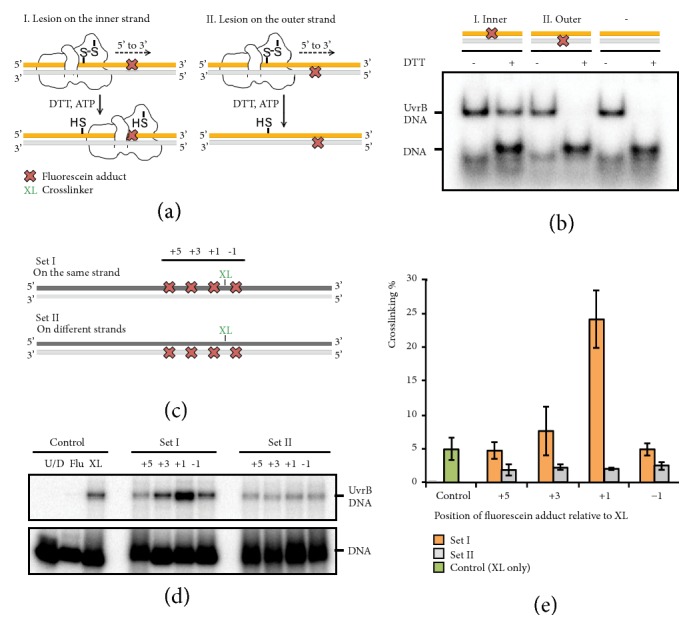
Biochemical assays identifying the lesion-containing strand. (a) Schematic illustrating the UvrB translocation assay. (b) The reactions analyzed by native PAGE. (c) 50-mer DNA substrates used for UvrB crosslinking assay. A single fluorescein-adducted thymine (Flu-dT) is located at various positions either on the same (Set I) or opposite (Set II) strand to a disulfide-bearing tether (XL). (d) Crosslinked products analyzed by SDS-PAGE. Control reactions contain the unmodified duplex DNA (U/D) and duplex DNA containing either fluorescein (Flu) or disulfide-bearing tether (XL) alone. (e) The average and standard deviation of three independent experiments is shown for each DNA substrate shown in Figure 6(c).

In order to provide additional biochemical confirmation of our assignment for the lesion-containing DNA strand, we incorporated a well-established UvrB substrate, fluorescein-adducted thymine (Flu-dT), into a duplex DNA disulfide-crosslinked to UvrB (T251C). The lesion was positioned downstream of the *β*-hairpin either on the inner or outer DNA strand. These poised complexes were then treated with DTT to release the enzyme from its staging point, and ATP was included to power duplex translocation (Figure 5(a)). Under these conditions, a persistent UvrB-DNA complex was observed only when the lesion was positioned on the inner strand, indicating that UvrB can recognize damage on the inner strand, but not the outer strand, while translocating along the inner strand in the 5′ to 3′ direction (Figure 5(b)). Next, we subjected UvrB(T251C) to a UvrA-assisted DNA binding assay with duplex DNA substrates containing a cystamine crosslinker (XL) and a Flu-dT at various positions either on the same strand or opposite strand (Figure 5(c) and Supplementary [Supplementary-material supplementary-material-1]). Although substrates amongst each set showed similar UvrB binding as judged by native PAGE, efficient crosslinking was observed only when the lesion was positioned on the 5′-side of the crosslinker on the same strand (Figures 5(d) and 5(e) and Supplementary Figures [Supplementary-material supplementary-material-1] and [Supplementary-material supplementary-material-1]). Since crosslinking is dependent upon extrusion of the XL nucleobase from DNA, these data are consistent with the notion that (i) UvrB catalyzes nucleobase extrusion and hence presumably bubble formation while scanning DNA for damage and (ii) the lesion-containing strand is the inner strand.

## 3. Discussion

Here we have presented the first structures of UvrB bound to duplex DNA in a manner that makes evident the salient features of lesion recognition and presentation of the bound lesion-containing duplex to UvrC. Even though the present structures do not formally contain a bona fide lesion, they exhibit the key structural attributes known from biochemical studies to be associated with lesion recognition: a bound DNA duplex containing a 6 bp bubble which presents the excised strand to the surface of UvrB and which positions the scissile phosphates for access and cleavage by UvrC. This being the case, the extrahelical, disulfide crosslinked nucleoside represents a simulated lesion and indeed is positioned thus with respect to the 3′- and 5′-incision sites.

**Figure 6:**
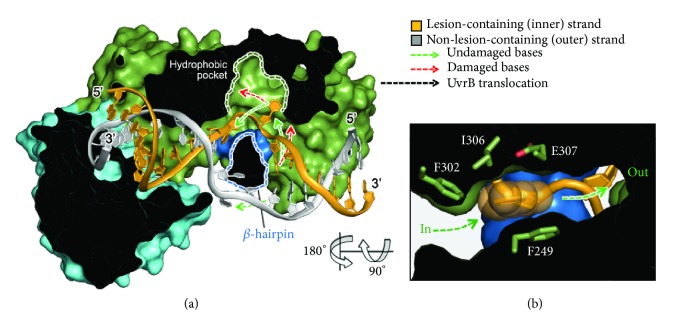
Proposed lesion recognition mechanism and biochemical studies. (a) The cross-section view of UvrB-dsDNA-ADP·Pi complex in surface representation. Proposed trajectories of undamaged and damaged “bulky” bases are shown in green and red arrows, respectively. Green and blue dashed lines delineate the boundaries of the lesion-selectivity filter and *β*-hairpin. (b) The cross-section view of the UvrB lesion-selectivity filter.

**Figure 7:**
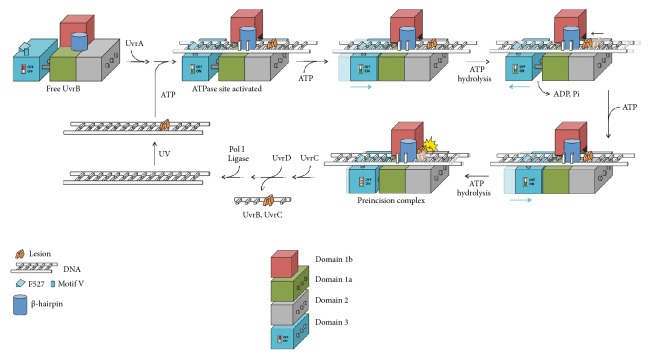
Proposed DNA repair mechanism in the Uvr nucleotide excision repair system.

The synthetic crystallography approach employed here conclusively establishes, for the first time, the identity of the lesion-containing strand and provides a compelling solution to the long-standing riddle of how UvrB recognizes such a diverse array of lesions. We propose that, following localization of UvrB by UvrA to a site nearby a lesion, UvrB unwinds the DNA, forming a 6 bp bubble that is flanked by F527 on one end and the *β*-hairpin at the other end. Fueled by ATP hydrolysis, the helicase activity of UvrB drives migration of the protein and the 6 bp bubble along the length of the DNA. During this migration, the lesion-containing strand passes through a hydrophobic constriction point through which a normal nucleobase can readily pass, but which poses an impediment to the passage of nucleobases bearing bulky appendages (e.g., an AAF adduct) or of lesions that bear a rigid connection between adjacent bases (e.g., a thymine dimer or cisplatin adduct) (Figures 6 and 7). Failure to transit past this constriction point would arrest UvrB translocation, leading to the formation of a preincision complex and subsequent recruitment of UvrC (Figure 7). F302 appears to serve as a gatekeeper for the UvrB lesion-selectivity filter, with F249, I306, and E307 forming the actual constriction point (Figure 6(b)). This selectivity filter is collapsed in the absence of a DNA substrate between the *β*-hairpin and domain 1b and only assumes the proper configuration only upon formation of a lesion-scanning complex (Supplementary [Supplementary-material supplementary-material-1]).

The utilization of a constriction-based lesion-recognition mechanism has been proposed previously [17, 18, 29], and the present structure provides strong structural support for those proposals. Based on the incision assay shown in Figure 2, we proposed that the nucleobase embedded in the hydrophobic pocket is recognized as a lesion-mimetic by UvrB, but the crosslinking assay (Figures 5(c)–5(e)) indicates that the base at the 3′-side of the fluorescein lesion is extrahelical. To resolve this discrepancy, we note that the UvrB selectivity filter is constricted in one dimension (Figure 6(b)) but wider and deeper in the other two dimensions (Figure 6(a)) than what is required to accommodate a single undamaged base. As predicted previously [46], this slotted character of the selectivity filter may permit entry of certain planar lesions but could prevent them from exiting owing to extensive hydrophobic interactions, such that the lesion becomes stuck within the selectivity filter while allowing extrusion of the nucleotide immediately 3′- to it. Extrusion of the base directly on the 3′-side of the fluorescein lesion is in agreement with previous biochemical studies that reported the base adjacent to the cholesterol- or menthol-adducted DNA on the 3′-side being extrahelical [29, 47]. Since NER pathway removes lesions as part of a nucleotide fragment by cleaving at a distance from the lesion, precise positioning of the lesion either within or immediately adjacent to the lesion-selectivity filter is not essential; both can enable efficient excision of the lesion.

## 4. Materials and Methods

### 4.1. Cloning, Overexpression, and Purification

B. caldotenax UvrA, UvrB, and UvrC proteins were cloned into pET-28a(+) vector (Novagen) and expressed in* E. coli* BL21 (DE3) pLysS using standard protocol. Harvested cells were lysed using sonication, followed by centrifugation at 35,000 g to clear the lysate. Subsequently, all proteins were purified using nickel-nitrilotriacetic acid (Ni-NTA) agarose (Qiagen), Heparin agarose (GE Healthcare), and size-exclusion chromatography using HiLoad 16/600 Superdex 200 (GE Healthcare). Cysteine residues for disulfide crosslinking (T251C or S91C) and a stop codon to generate domain 4 deletion construct (Δ594-658) were introduced to the UvrB expression vector using a QuikChange II site-directed mutagenesis kit (Agilent Technologies). To obtain homogeneous UvrB-DNA disulfide-crosslinked complexes, all native cysteine residues in B. caldotenax UvrB (C144, C211, and C303) were mutated to serine for both structural and biochemical studies. Only for crystallographic studies, His-tag was cleaved from UvrB by overnight incubation with TEV protease at 4°C prior to size-exclusion chromatography. UvrA and UvrC were frozen in 25 mM Tris pH 7.4, 250 mM NaCl, 20% (v/v) glycerol, and 5 mM *β*ME at -80°C until use. UvrB was stored in the same buffer but was lacking *β*ME.

### 4.2. Oligonucleotide Synthesis and Purification

All site-specifically modified single-stranded oligonucleotides were synthesized on ABI 392 DNA synthesizer (Applied Biosystems) using standard protocol and reagents in 1 *μ*M scale, purified by urea-PAGE, and desalted by Sep-Pak columns (Waters). Oligonucleotides containing a disulfide-bearing tether at the N^4^-position of a cytosine or at the nonbridging position of DNA backbone were synthesized and functionalized as described previously [48, 49]. Oligonucleotide modification was confirmed by MALDI-TOF. All unmodified oligonucleotides were purchased from Eurofins Genomics.

### 4.3. Crosslinking and Purification of UvrB-dsDNA Complexes

For assembly of UvrB(T251C)-dsDNA complexes for crystallization, a single-stranded 20-mer oligonucleotide containing a thiol-tether attached to a cytosine (100 *μ*M, 5′-GCTCTAGATTTTC*∗*ATACGGC-3′, where C*∗* denotes a disulfide-tethered cytosine) was mixed with a truncated UvrB mutant (40 *μ*M, Δ594-658) in 25 mM Tris pH 7.4, 200 mM NaCl, at 16°C for 3-5 days. UvrB disulfide-crosslinked to a single-stranded oligonucleotide was purified by Mono-Q anion exchange chromatography (GE Healthcare) using a linear salt gradient of 95% Buffer A (25 mM Tris pH 7.4) to 25% Buffer B (25 mM Tris pH 7.4, 2.0 M NaCl). Fractions containing the complex were pooled and mixed with 1.5 molar excess of the complementary oligonucleotide containing two pyrimidine-pyrimidine mismatches (5′-GCCGTATGCCAATCTAGC-3′, where mismatched bases are underlined) for annealing at 4°C for 18 hrs. UvrB(S91C)-dsDNA and UvrB(S91C/F527A)-dsDNA complexes were obtained similarly by assembling 16-mer oligonucleotides (5′-TCTCCATCG_CGCTACC-3′, where the underlined position indicates the site of backbone modification to a phosphoramidite with a cystamine and 5′-GGTAGCGCGATGGAGA-3′). Purification of UvrB disulfide-crosslinked to the duplex DNA was performed on a Mono Q using a linear salt gradient of 95% Buffer A to 30% Buffer B, followed by size exclusion chromatography on Superdex75 10/300 (GE Healthcare) using buffer (25 mM Tris pH 8.0, 150 mM NaCl).

### 4.4. Crystallization, Data Collection, and Structure Determination

All crystals were obtained by the sitting-drop vapor diffusion method at 20°C. UvrB(T251C)-dsDNA complex was crystallized in 100 mM Bis-Tris (pH 6.5), 100 mM ammonium acetate, and 17-19% (w/v) polyethylene glycol (PEG) 8000 at 6.5 mg/ml complex concentration. Crystals of UvrB(T251C)-dsDNA bound to ADP and Pi were grown in similar conditions, except the reservoir that contained 2 mM ATP and 5 mM MgCl_2_. UvrB(S91C)-dsDNA-ADP crystals were obtained in 0.1 M HEPES pH 7.5, 0.02 M MgCl_2_, and 20-22% poly(acryl acid sodium salt) 5100 at 5 mg/ml complex concentration. UvrB(S91C/F527A)-dsDNA crystals were grown in 0.2 M potassium sulfate, 18-20% PEG3350, 2 mM ADP, and 5 mM MgCl_2_ at 5 mg/ml complex concentration, but the electron density corresponding to ADP was not observed. All crystals were transferred to a reservoir solution supplemented with 20% (v/v) glycerol and then frozen in liquid nitrogen for data collection. Diffraction datasets were collected at -170°C at the 24-ID-C and 24-ID-E beamlines (NE-CAT) of the Advanced Photon Source. Datasets were processed with the HKL program suites [50]. Initial molecular replacement solutions were obtained by PHASER in the CCP4 suite [51], using the coordinates of previously determined UvrB structures as search models. UvrB-dsDNA models were built through iterative cycles of manual model building in COOT [52] and structure refinement using REFMAC5 [53] and PHENIX [54]. The Ramachandran plots, calculated by MolProbity [55], contain no residues in disallowed regions for all structures. All the structure model figures in the paper were prepared using PyMol (The PyMOL Molecular Graphics System, Version 1.3, Schrödinger, LLC.). Full details on the data collection and structure refinement are contained in Supplementary [Supplementary-material supplementary-material-1]. The coordinates and structure factors have been deposited in the Protein Data Bank under accession codes 6O8E, 6O8F, 6O8G, and 6O8H.

### 4.5. UvrC Incision Assay

UvrB(T251C)-dsDNA complexes for UvrC incision assays were prepared via disulfide-crosslinking and stepwise strand assembly using a crosslinker-containing 32-mer single-stranded oligonucleotide (5′-TATGATGGAGACTCTTTTTTC*∗*GCGGCAATTTT-3′, where C*∗* denotes a disulfide-tethered cytosine) and 32-mer (5′-TAAAATTGCCGCGAAAAAAGAGTCTCCATCAT-3′) complementary oligonucleotides. In order to label both strands of the duplex DNA bound to UvrB(T251C), 10 fmol of the purified UvrB(T251C)-dsDNA complexes were added to 1 *μ*Cu *γ*-[^32^P]-ATP (7,000 Ci mmol^−1^, Perkin Elmer) and 5 units of T4 polynucleotide kinase in 1X PNK buffer (New England Biolabs) for 1 hour at 37°C. The 5′ end labeled UvrB-dsDNA complexes (2 nM) were then incubated with 5-100 nM UvrC in 50 mM Tris pH 7.4, 100 mM NaCl, 0.1 mg/ml BSA, 1 mM ATP, 10 mM MgCl_2_, and 5% (v/v) glycerol at 55°C for 30 minutes. Reactions were terminated by adding an equal volume of a stop buffer (20 mM EDTA, 95% formamide, 0.05% bromophenol blue, and 0.05% xylene cyanol). The reaction mixtures were analyzed on 15% or 23.5% denaturing polyacrylamide gels and visualized via phosphor-imaging using a Typhoon phosphor imager and ImageQuant software (GE Healthcare).

### 4.6. UvrB Translocation Assay

UvrB(T251C) bound to a DNA duplex containing a single fluorescein-adducted thymine on the inner strand (5′ TACCCCGTTGCATCC*∗*TAG(Flu-dT)TCCCCAAAATTTT 3′ paired with 5′-AAAATTTTGGGGAACTAGGATGCAACGGGGTA-3′, where C*∗* and Flu-dT denote a disulfide-tethered cytosine and fluorescein-adducted thymine, resp.), DNA duplex containing a fluorescein lesion on the outer strand (5′ TACCCCGTTGCATCC*∗*TAGATCCCCAAAATTTT 3′ paired with 5′-AAAATTTTGGGGT(Flu-dT)FCTAGGATGCAACGGGGTA-3′), or DNA duplex containing no lesion (5′ TACCCCGTTGCATCC*∗*TAGTTCCCCAAAATTTT 3′ paired with 5′-AAAATTTTGGGGAACTAGGATGCAACGGGGTA-3′) were prepared via disulfide crosslinking and stepwise strand assembly and 5′ ends labeled as described above. Complexes (2 nM) were then incubated in 50 mM Tris pH 7.4, 100 mM NaCl, 0.1 mg/ml BSA, 1 mM ATP, 10 mM MgCl_2_, 5% (v/v) glycerol, and 5 mM DTT for 15 minutes at 55°C. The reaction products were analyzed on 6% native polyacrylamide gel containing 1 mM ATP and 10 mM MgCl_2_ and visualized via phosphor-imaging using a Typhoon phosphor imager and ImageQuant software (GE Healthcare).

### 4.7. Crosslinking Assay

50-mer single-stranded oligonucleotides containing a thiol-tether (Supplementary [Supplementary-material supplementary-material-1]) were radioactively labeled at 5′ ends and then annealed with their respective complementary oligonucleotides in a 1.2-fold molar excess in buffer (10 mM Tris pH 8.0). The labeled duplex substrates (2 nM) were incubated with 2.5 nM UvrA, 100 nM UvrB in 50 mM Tris pH 7.4, 100 mM NaCl, 0.1 mg/ml BSA, 1 mM ATP, 10 mM MgCl_2_, and 5% (v/v) glycerol for 30 minutes at 55°C. Each reaction mixture was split into two and analyzed by both 6% native-PAGE and 6% SDS-PAGE in nonreducing conditions and visualized as describe above. The relative radioactivities of the bands were evaluated employing the ImageQuant software (GE Healthcare). All reactions were completed in triplicate and the data are presented as average ± standard deviation.

## Supplementary Materials

Supplementary Materials Figure S1: The location of the helicase motifs in the RecA-like domains of UvrB. Figure S2: Interactions between DNA and the *β*-hairpin. Figure S3: Comparison of UvrB-dsDNA complex to other SF2 helicases. Figure S4: Crystal structures of UvrB bound to a fully duplex DNA. Figure S5: Crosslinking assay to identify the lesion-binding site. Figure S6: UvrB lesion-selectivity filter. Table S1: Data collection and refinement statistics.
