# Supplementary material for: Mechanism of DNA Lesion Homing and Recognition by the Uvr Nucleotide Excision Repair System
Source: Research (Wash D C). 2019 Aug 28;2019:5641746. doi: 10.34133/2019/5641746 (PMC6750098; doi:10.34133/2019/5641746)
Supplement: Supplementary Materials — Figure S1: The location of the helicase motifs in the RecA-like domains of UvrB. Figure S2: Interactions between DNA and the β-hairpin. Figure S3: Comparison of UvrB-dsDNA complex to other SF2 helicases. Figure S4: Crystal structures of UvrB bound to a fully duplex DNA. Figure S5: Crosslinking assay to identify the lesion-binding site. Figure S6: UvrB lesion-selectivity filter. Table S1: Data collection and refinement statistics. [file 5641746.f1.docx]

**
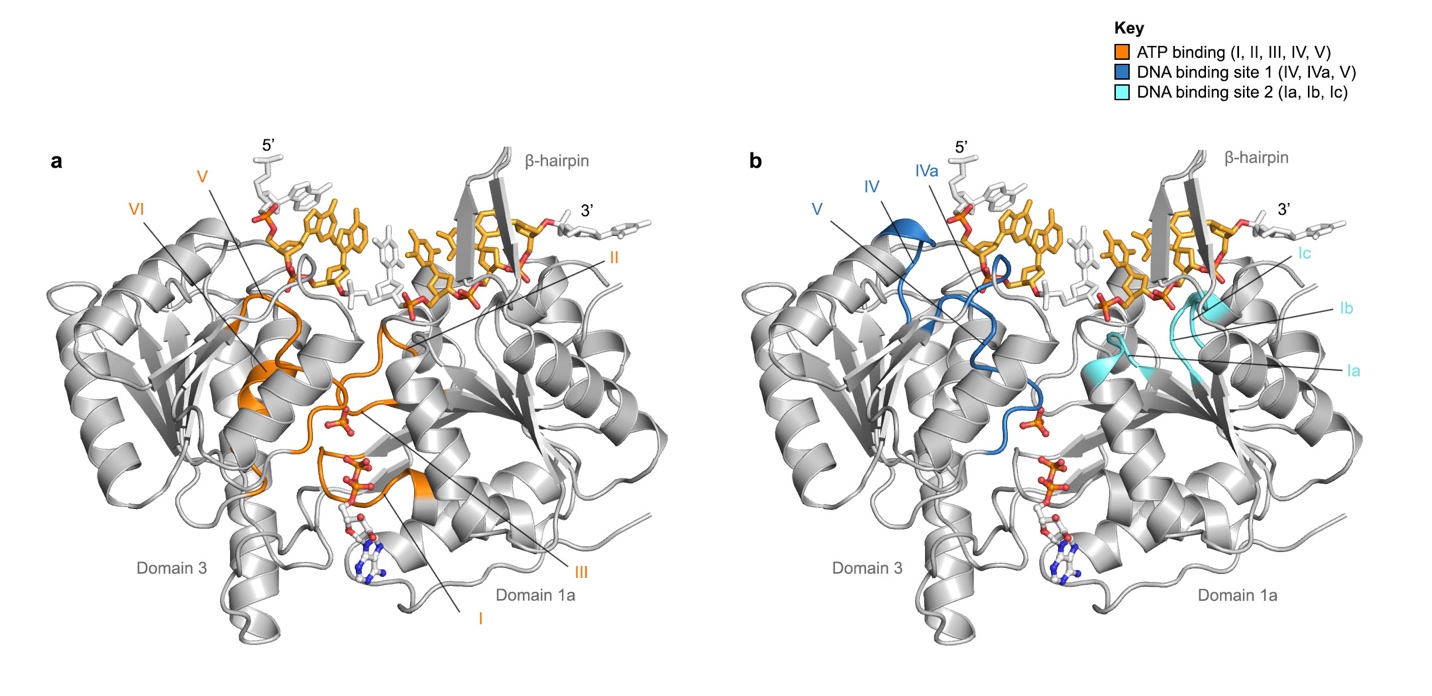
**

**Figure S1** | The location of the helicase motifs in the RecA-like domains of UvrB. **a,** Helicase motifs involved in ATP binding (orange). **b,** Helicase motifs involved in DNA binding (blue and cyan)**.** For clarity, only 8-nucleotide stretch of the inner strand and two RecA-like domains are shown in the figure. Nucleotides in contact with helicase motifs are shown in colors while others are shown in white.

**
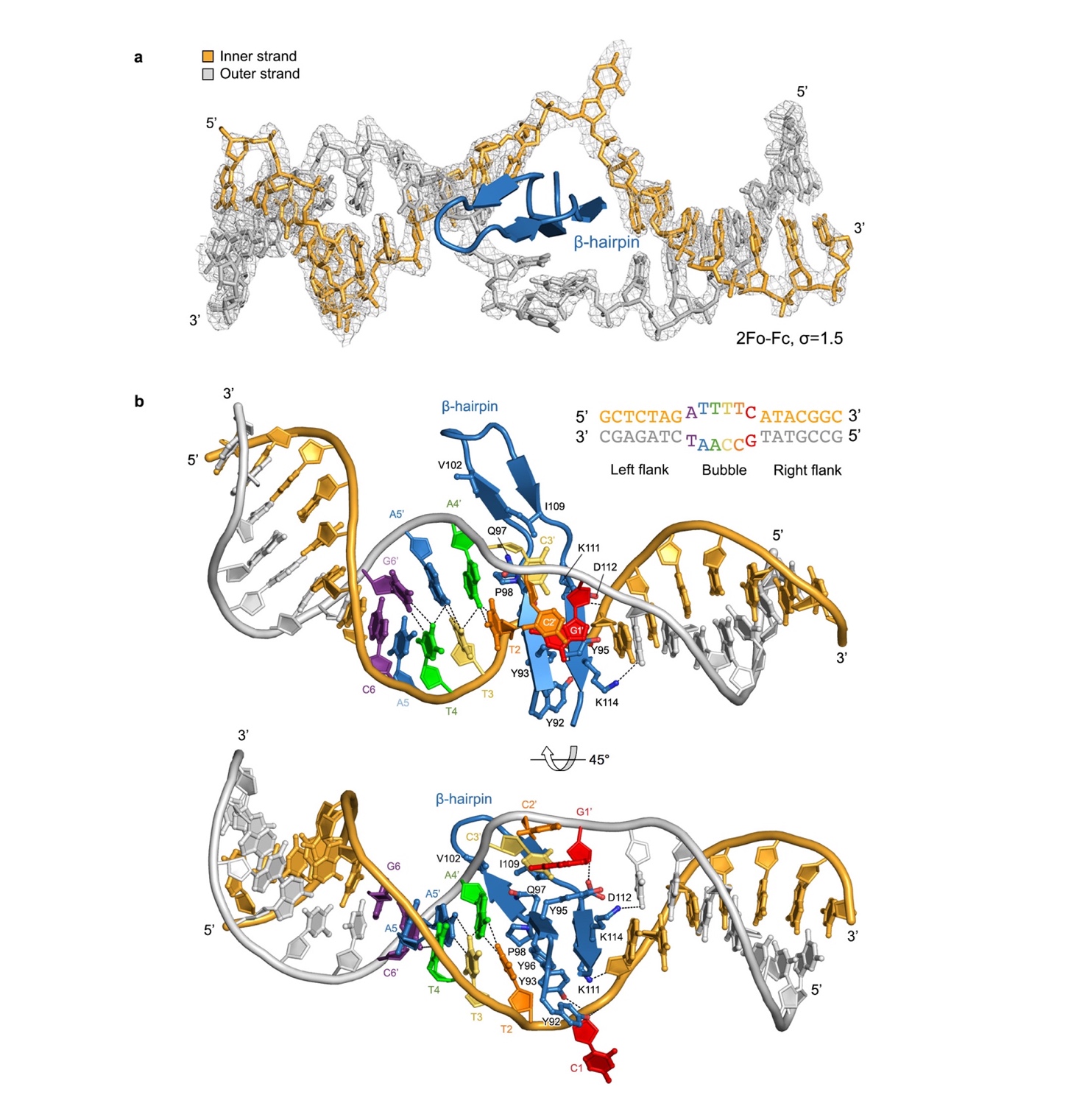
**

**Figure S2** | Interactions between DNA and the **β-**hairpin**. a**, 2Fo – Fc electron density map (grey mesh) contoured at 1.5 sigma covering the entire stretch of 20-mer duplex DNA. The inner and outer strands are shown in gold and grey, respectively, and the β-hairpin is shown in blue. **b**. Detailed views rotated by ~ 45°. The unpaired bases within the 6 bp bubble are shown in rainbow colors. Interactions are represented in dashed lines.

**
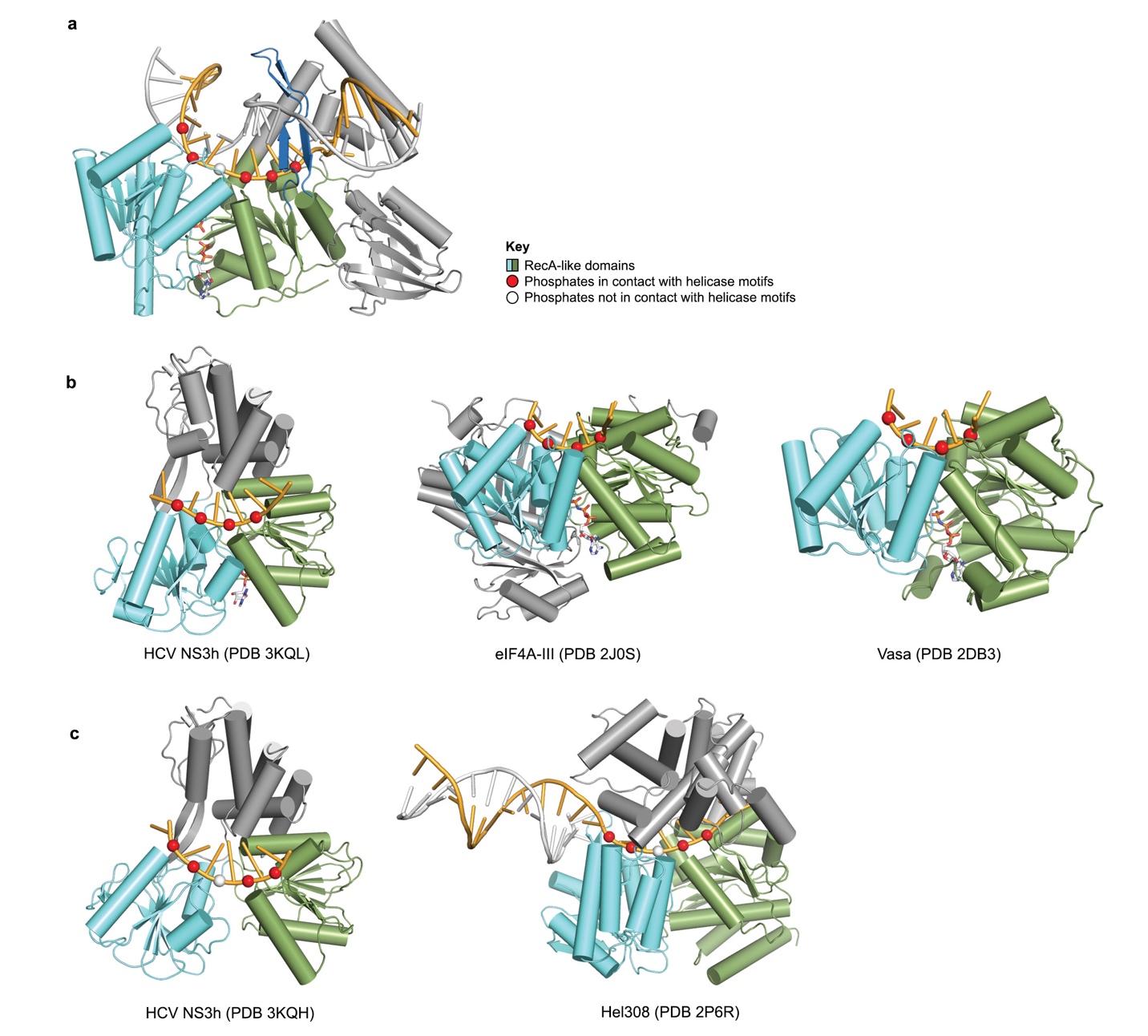
**

**Figure S3** | Comparison of UvrB-dsDNA complex to other SF2 helicases. **a,** Structure of the UvrB-dsDNA-ADP·Pi complex. **b,** SF2 helicase structures containing RecA-like domains in the closed state; Hepatitis C virus NS3 helicase[39] (PDB ID, 3KQL), eIF4A-III[41] (PDB ID, 2J0S), and Drosophila Vasa[40] (PDB ID, 2DB3), all of which are bound to a non-hydrolyzable ATP-analog. **c,** SF2 helicase structures containing RecA-like domains in the open state; Hepatitis C virus NS3 helicase[39] (PDB ID, 3KQH), and archaeal Hel308[34] (PDB ID, 2P6R). DNA backbone phosphates that make interactions with helicase motifs are shown in red spheres, while those not in contact are shown in white spheres.

**
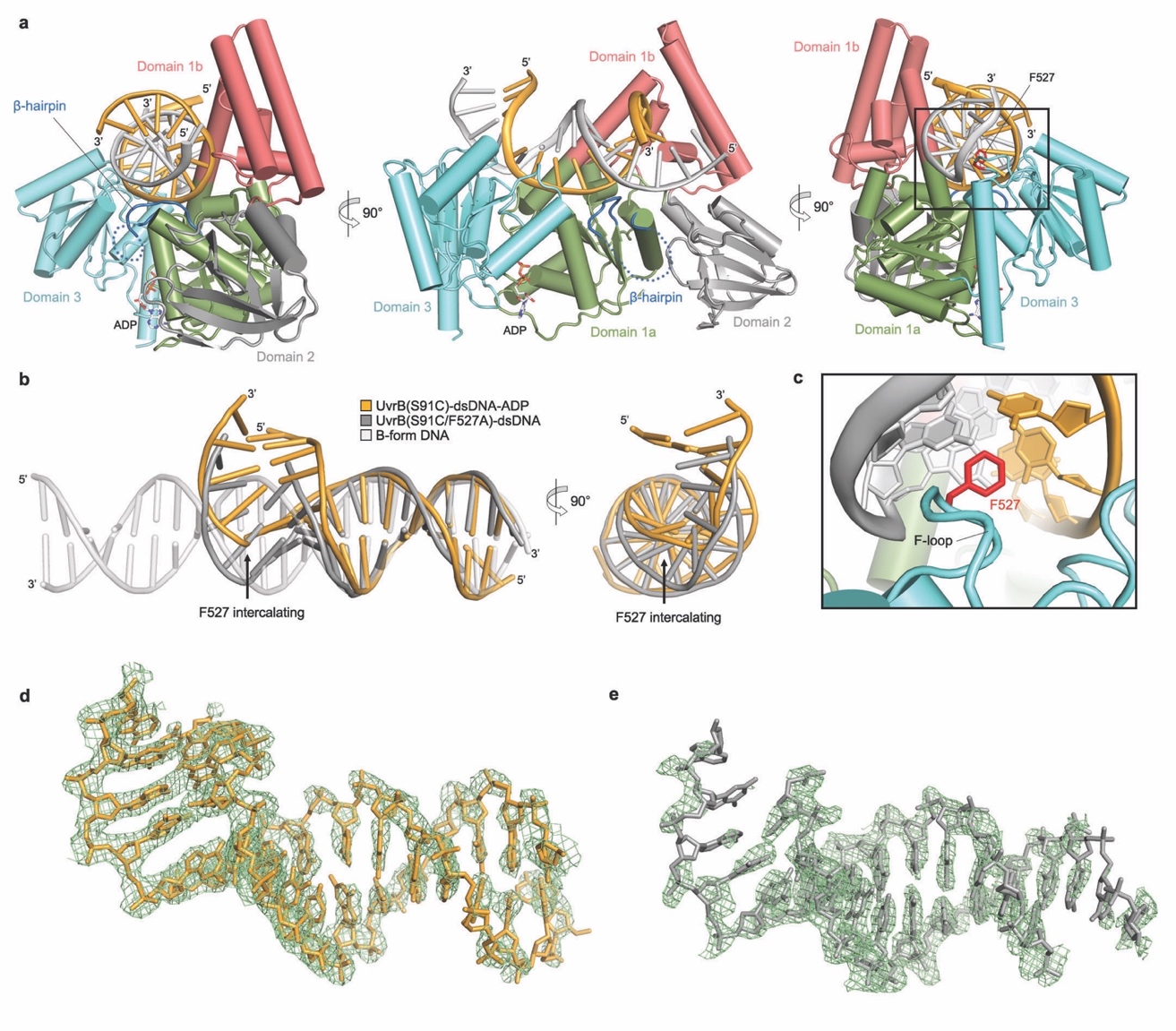
**

**Figure S4** | Crystal structures of UvrB bound to a fully duplex DNA. **a**, Overall structure of UvrB(S91C)-dsDNA-ADP complex in three views rotated by ~90°. UvrB domains 1a, 1b, 2, and 3 are coloured in green, pink, grey and cyan, respectively. The partially ordered β-hairpin that is not inserted between the two DNA strands is shown in blue. ADP and F527 side chain are shown in sticks. **b**, Duplex DNA bound to UvrB(S91C) and UvrB(S91C/F527A) in two orthogonal views. As a reference, an ideal B-form DNA is superimposed. **c**, Close-up view of F527 side chain (red) intercalating into the duplex stack. **d.** Fo – Fc omit electron density map (green mesh) contoured at 2 σ covering the entire 16-mer duplex DNA tether to UvrB(S91C). **e.** Fo – Fc omit electron density map (green mesh, 2 σ) tethered to UvrB(S91C/F527A), showing DNA disorder on both ends of the duplex.


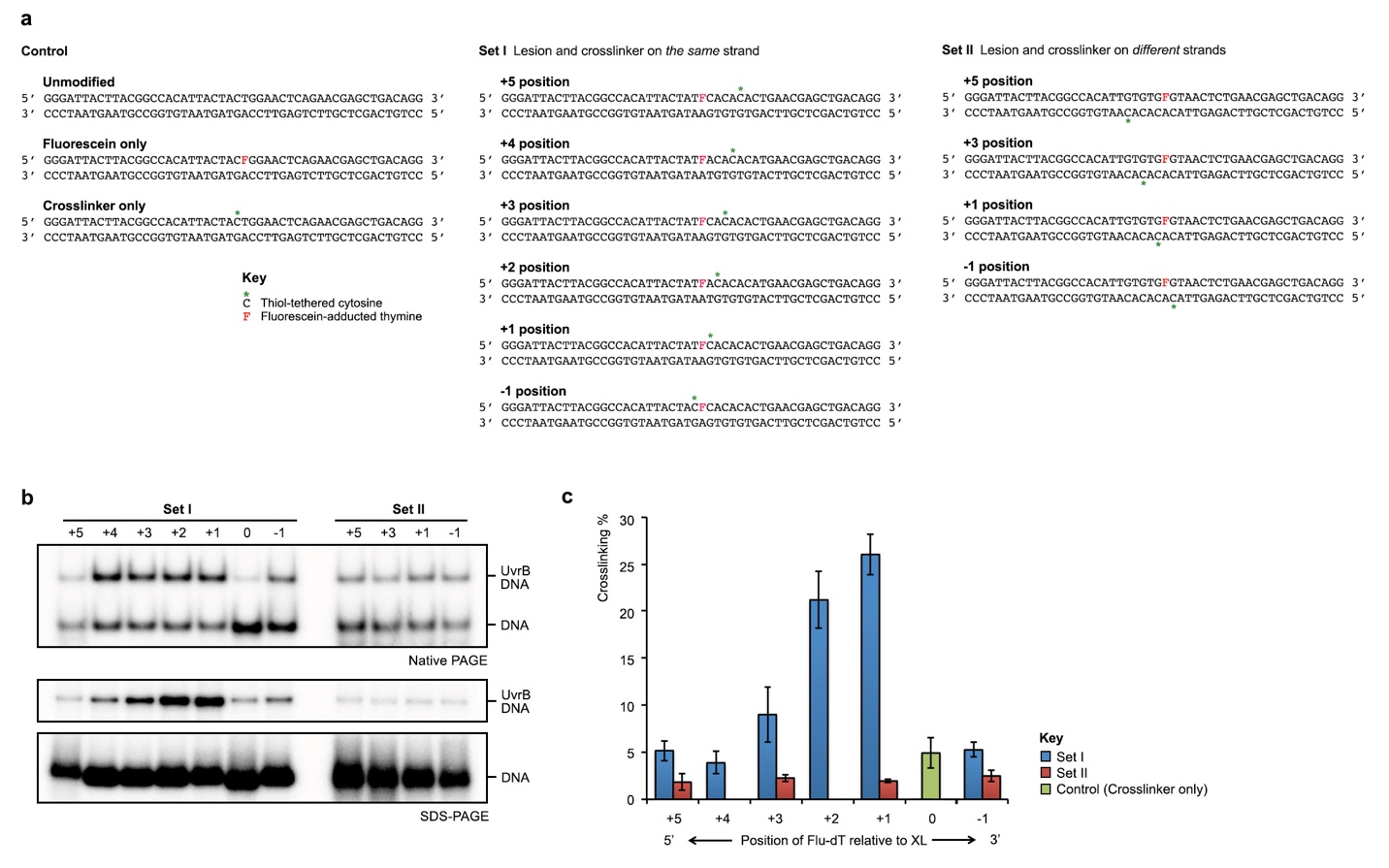


**Figure S5** | Crosslinking assay to identify the lesion-binding site. **a,** 50-mer DNA substrates used for UvrB crosslinking assay. Thiol-tether (XL) containing strands are radioactively labeled at 5’ ends. A single fluorescein-adducted thymine is located at various positions either on the same strand as or on the opposite strand to a thiol-tether.  **b,** After incubating with UvrA, UvrB(T251C) and ATP, the reaction mixtures were analyzed by 6% native PAGE (top) and 6% SDS-PAGE (bottom). **c,** Crosslinking yield of the DNA substrates shown in **a**. Error bars reflect the standard deviation of the mean in triplicate.


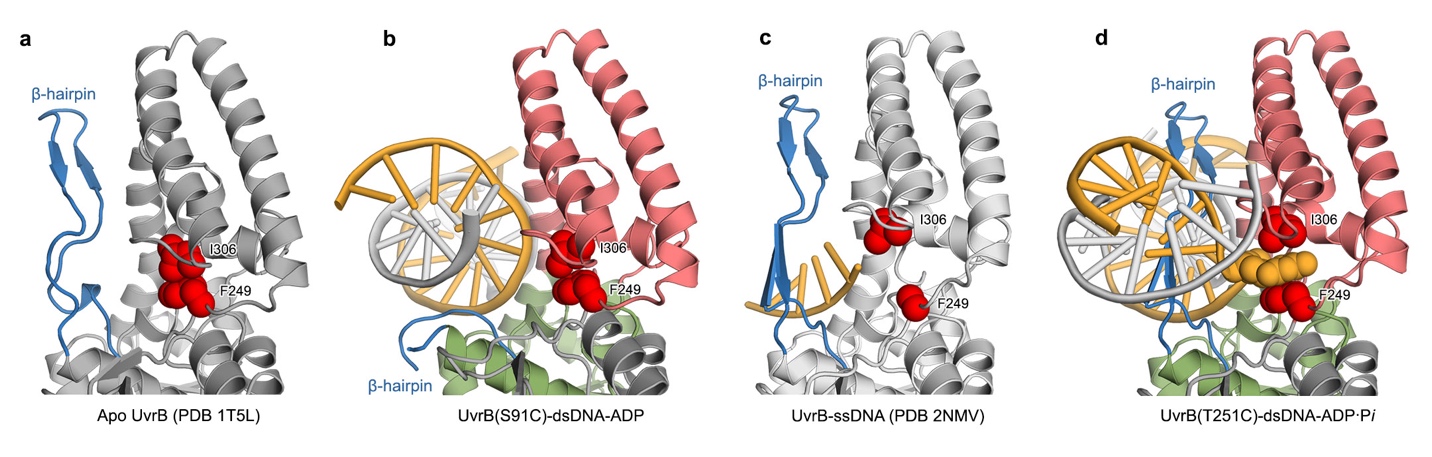


**Figure S6** | UvrB lesion-selectivity filter. **a,** The constriction point formed by I306 and F249 (red) is collapsed in the absence of a DNA substrate (PDB ID, 1T5L)^14^. **b,** The constriction point is collapsed when the β-hairpin is uninserted as in UvrB(S91C)-dsDNA-ADP. **c**, The lesion-selectivity filter is open when a DNA substrate is bound between the β-hairpin and domain 1b (PDB ID, 2NMV)^16^. **d**, The properly configured lesion-selectivity filter in the UvrB-dsDNA-ADP·Pi complex. The extruded cytosine inserted into the hydrophobic pocket is shown in spheres.

**Table S1** | Data collection and refinement statistics

|  | UvrB(T251C)  -dsDNA-ADP·Pi | UvrB(T251C)  -dsDNA | UvrB(S91C)  -dsDNA-ADP | UvrB(S91C/F527A)  -dsDNA |
| --- | --- | --- | --- | --- |
| **Data collection** |  |  |  |  |
| Space group | P22_1_2_1_ | P2_1_ | P2_1_2_1_2 | P4_1_2_1_2 |
| Cell dimensions |  |  |  |  |
| a, b, c (Å) | 69.3, 115.7, 226.8 | 58.2, 265.2, 68.3 | 170.7, 201.2, 62.7 | 124.8, 124.8, 96.2 |
| ɑ, β, 𝛾 (°) | 90, 90, 90 | 90, 114.4, 90 | 90, 90, 90 | 90, 90, 90 |
| Resolution (Å) | 70.5-2.61  (2.67-2.61) | 132.6-2.81  (2.91-2.81) | 49.52-2.63  (2.78-2.63) | 50.0-2.40  (2.49-2.40) |
| R_sym_ or R_merge_ | 0.083 (1.469) | 0.145 (0.980) | 0.055 (0.69) | 0.087 (0.62) |
| I/σI | 11.8 (0.7) | 9.1 (1.5) | 15.2 (1.8) | 18.9 (2.3) |
| Completeness (%) | 98.6 (99.5) | 99.8 (99.8) | 99.5 (99.7) | 99.8 (99.6) |
| Redundancy | 3.4 (3.5) | 3.8 (3.8) | 4.0 (4.1) | 6.2 (5.9) |
| CC_1/2_ | 0.998 (0.584) | 0.990 (0.553) | 0.996 (0.518) | 0.994 (0.534) |
|  |  |  |  |  |
| **Refinement** |  |  |  |  |
| Resolution (Å) | 70.5-2.61 | 132.6-2.81 | 48.2-2.64 | 48.3-2.39 |
| No. reflections | 61517 | 43235 | 61005 | 28828 |
| R_work/_ R_free_ | 0.215/0.270 | 0.217/0.268 | 0.220/0.274 | 0.227/0.277 |
| No. atoms |  |  |  |  |
| Protein | 9560 | 9567 | 13662 | 4622 |
| DNA | 1620 | 1620 | 1874 | 531 |
| Ligand/ion | 83 | 9 | 83 | 12 |
| Water | 16 | 32 | 26 | 158 |
| B-factors |  |  |  |  |
| Protein | 71.1 | 45.8 | 68.9 | 51.0 |
| DNA | 82.0 | 47.3 | 78.4 | 98.1 |
| Ligand/ion | 78.9 | 48.9 | 89.3 | 51.9 |
| Water | 47.8 | 29.0 | 53.1 | 44.9 |
| R.m.s deviations |  |  |  |  |
| Bond lengths (Å) | 0.010 | 0.010 | 0.005 | 0.005 |
| Bond angles (º) | 1.394 | 1.264 | 0.995 | 1.049 |
| PDB ID | 6O8E | 6O8F | 6O8G | 6O8H |

*Highest resolution shell is shown in parenthesis.
